# Wireless Magnetoelectrochemical Induction of Rotational Motion

**DOI:** 10.1002/advs.202306635

**Published:** 2023-12-21

**Authors:** Kostiantyn Tieriekhov, Neso Sojic, Laurent Bouffier, Gerardo Salinas, Alexander Kuhn

**Affiliations:** ^1^ University of Bordeaux CNRS Bordeaux INP ISM, UMR 5255 Talence 33400 France

**Keywords:** bipolar electrochemistry, Lorentz force, magnetohydrodynamics, rotors

## Abstract

Electromagnetically induced rotation is a key process of many technological systems that are used in daily life, especially for energy conversion. In this context, the Lorentz force‐induced deviation of charges is a crucial physical phenomenon to generate rotation. Herein, they combine the latter with the concept of bipolar electrochemistry to design a wireless magnetoelectrochemical rotor. Such a device can be considered as a wet analog of a conventional electric motor. The main driving force that propels this actuator is the result of the synergy between the charge‐compensating ion flux along a bipolar electrode and an external magnetic field applied orthogonally to the surface of the object. The trajectory of the wirelessly polarized rotor can be controlled by the orientation of the magnetic field relative to the direction of the global electric field, producing a predictable clockwise or anticlockwise motion. Fine‐tuning of the applied electric field allows for addressing conducting objects having variable characteristic lengths.

## Graphical abstract

An electrochemical analog of an electric motor is presented here as a new concept to generate the rotational motion of a conducting object based on the synergy between bipolar electrochemistry and an external magnetic field.

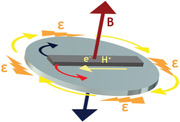

## 1 Introduction

The interconversion of a physicochemical phenomenon into kinetic energy is one of the most fundamental concepts of science since it is a crucial ingredient of numerous technological applications, e.g. energy conversion, propulsion, or pumping. Different approaches for the generation of motion, involving gradients of surface tension (Marangoni effect),^[^ ^1^, ^2^, ^3^, ^4^ ^]^ chemical composition^[^ ^5^ ^]^ and temperature,^[^ ^6^ ^]^ or external stimuli such as light,^[^ ^7^, ^8^ ^]^ sound^[^ ^9^, ^10^ ^]^ or electric^[^ ^11^, ^12^ ^]^ and magnetic fields,^[^ ^13^, ^14^, ^15^, ^16^, ^17^, ^18^ ^]^ have been explored and are practically used. In this context, the electromagnetic induction of rotation is the basis of many devices used in daily life, such as electric motors, generators, or alternators. Commonly in these systems, rotation is generated by the continuous self‐alignment between electromagnetic fields. However, such devices require direct electric connections, limiting their application, especially at small length scales. Therefore, investigating alternative systems, allowing a wireless electromagnetic induction of rotation, is very interesting, not only from a purely academic point of view, motivated by the demonstration of a set proof‐of‐principle experiments, but also in view of future applications, such as integrating intrinsic stirring mechanisms into electrochemical experiments, either for analytical systems or for enhancing the performance of electrosynthetic processes via an enhanced mass transport.

An attractive concept is to take advantage of the Lorentz force ($\vec{F_{L}}$), induced by the presence of an external magnetic field ($\vec{B}$) and a flux of charges.^[^ ^19^, ^20^ ^]^ It is well‐established that by placing a current‐carrying wire orthogonal to $\vec{B}$, the flux of electrons experiences a force perpendicular to the direction of the current and the magnetic field. The same electromagnetic effect can also be observed for an ion flux generated around an electrode. This phenomenon has been extensively used to improve mass transport limitations associated with electrodeposition, electrocatalysis, and microfluidic systems.^[^ ^21^, ^22^, ^23^, ^24^, ^25^, ^26^ ^]^ Recently the concept has also been successfully exploited to boost the kinetics of self‐electrophoretic devices.^[^ ^27^, ^28^, ^29^ ^]^ In this case, a potential difference (*ΔV*) is induced between the two extremities of a conducting object by coupling thermodynamically spontaneous redox reactions occurring at each end of the device. Such self‐polarized objects exhibit predictable clockwise or anticlockwise motion as a function of the orientation of the magnetic field. A promising alternative is to induce the asymmetric polarization of an object by using bipolar electrochemistry (BE). In this case, applying a high enough external electric field (*ε*) allows the thermodynamic threshold polarization potential (*ΔV*_min_), defined by two coupled nonspontaneous redox reactions, to be overcome. This concept has been successfully used to asymmetrically trigger redox reactions in the frame of multiple electrochemical processes involved in sensing, organic synthesis and chemical separation.^[^ ^30^, ^31^, ^32^, ^33^, ^34^ ^]^ In addition, bipolar electrochemical rotation based on a bubble accumulation/release mechanism or by generating an asymmetric charge‐compensating proton flux along the object has been developed.^[^ ^12^, ^35^, ^36^ ^]^ However, such dynamic systems require the design of rather complex bipolar electrodes (BPEs).

In the present work, we take advantage of the synergy of BE and the Lorentz force to design a wireless magnetoelectrochemical rotor as a wet analog of a classic electric motor. By coupling the oxidation of hydroquinone (HQ) and reduction of benzoquinone (Q), respectively, a continuous charge‐compensating proton flux is generated along the BPE. Thus, in the presence of a magnetic field, positioned orthogonally to the surface of the device, the induced Lorentz force produces a macroscopic flux of fluid, which propels the polarized object. Furthermore, the interplay between the direction of the applied electric field and the orientation of the external magnetic field allows a controllable clockwise or anticlockwise full rotation to be generated. To the best of our knowledge, this is the first example of such a freely rotating device driven by simple electrochemical reactions.

## 2 Results

### 2.1 Concept of Magnetoelectrochemical Rotation

**Scheme 1:**
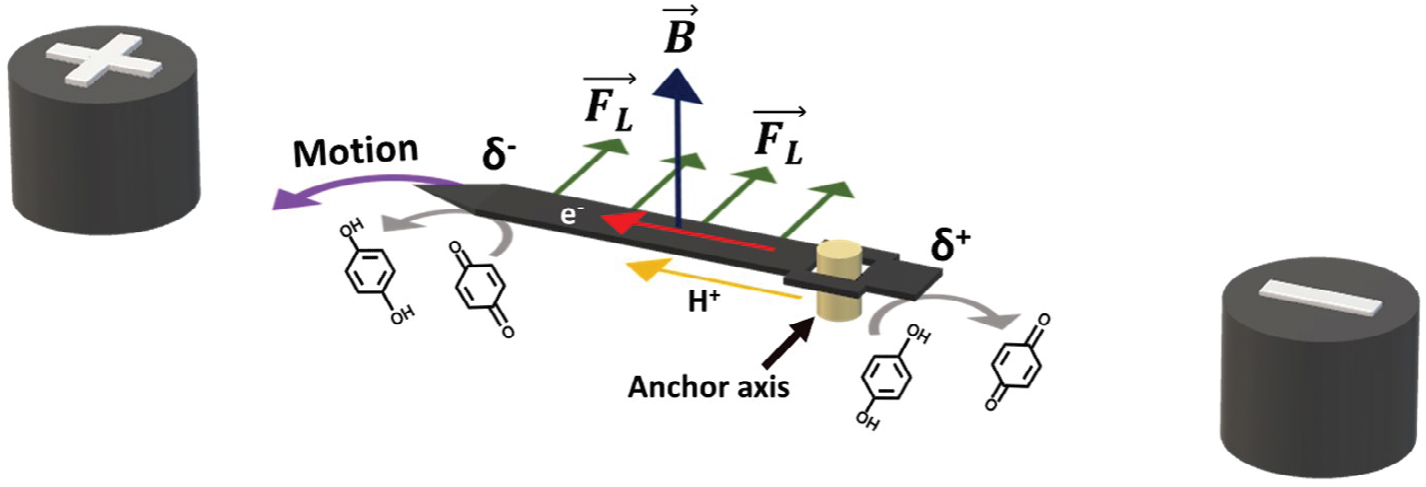
Schematic illustration of the wireless induction of rotation of a carbon strip acting as a bipolar electrode (BPE) positioned between two feeder electrodes. The anchor axis is located at the anodic pole of the device, with a representation of the associated electrochemical reactions, the anodically and cathodically polarized extremities (*δ^+^* and *δ^−^*, respectively), the electric and charge‐compensating ionic currents, the magnetic field ($\vec{B}$) and the resulting horizontal Lorentz force ($\vec{F_{L}}$).

The anisotropic rotors were designed by cutting carbon foil in rectangular shapes with different lengths (*l*). In order to improve the conductivity of the BPEs, a thin gold layer was deposited on top of the carbon strips. The dynamic behavior of the rotors was evaluated by placing the object at the center of a conventional bipolar cell, with the gold‐modified face in contact with the air/water interface of a 5 mm LiClO_4_/1 µm sodium dodecyl benzenesulfonate (DBS) solution, containing both Q and HQ redox fuels in an equimolar ratio (5 mm). The bipolar cell was placed at the center of a rectangular FeNdB magnet (*B* ≈ 200 mT) with a surface area (*A*) of 98 cm^2^, and the north pole facing upward (see blue arrow in **Scheme** **1**). The same experimental setup was used for all the measurements unless otherwise indicated. As stated above, when a high enough electric field is applied between the feeder electrodes, an asymmetric polarization of the BPE occurs, inducing the oxidation and reduction of HQ and Q, respectively (Scheme 1). These redox reactions generate a charge‐compensating proton flux from the anode to the cathode of the BPE. However, in order to produce a rotational displacement, two types of additional symmetry breaks are required. The first one originates from the presence of the external magnetic field orthogonal to the BPE surface. Under these conditions, a Lorentz force is induced on the charge‐compensating ion flow, accompanied by a lateral flux of liquid, pushing the device in the opposite direction (Scheme 1). The second break of symmetry is related to the position of the anchor axis along the BPE. By placing the anchor as close as possible to one of the extremities of the polarized object, the magnitude of the torque force that induces rotation is maximized (Scheme 1). It is noteworthy that, besides the above‐mentioned forces, a macroscopic magnetohydrodynamic (MHD) vortex is generated between the feeder electrodes (Scheme S1, Supporting Information). This is due to the induction of a Lorentz force on the electrolyte charges moving between the feeder electrodes, orthogonal to the external $\vec{B}$. The intensity and direction of such a macro‐MHD vortex is a function of the applied *ε* and the orientation of the magnetic field.

### 2.2 Magnetoelectrochemical Induction of Motion

To test the above‐described wireless magnetoelectrochemical propulsion mechanism and to evaluate the influence of the macro‐MHD vortex, a set of control experiments has been carried out using four different objects with a length of 1.5 cm; two completely insulating plastic devices and two carbon foil BPEs. For both materials two different positions of the anchor were evaluated; the anchor axis was located either at the center or at the anodic extremity of the devices. The dynamic behavior of each device was evaluated by applying a constant *ε* value (5 V cm^−1^) for 30 s. As expected, the plastic rotor with the anchor axis at the center remains static in the time frame of the experiment, whereas the plastic device with the anchor located at the extremity facing the feeder cathode, as well as the symmetric carbon BPE (anchor at the center), exhibit a slight clockwise rotation (Figure S1a−c; Video S1, Supporting Information). This is consistent with the theoretical direction of the macro‐MHD vortex of the liquid predicted by the right‐hand rule. On the contrary, when using the carbon foil with an asymmetric anchor position, the corresponding redox reactions, taking place at the anode and cathode of the device, trigger an instantaneous rotation (Figure S1d; Video S1, Supporting Information). This motion, opposite to the macro‐MHD vortex, is attributed to the synergy between the induced Lorentz force on the charge‐compensating proton flux and the spatial distribution of the torque force along the axis of the rotor. The direction of the vector $\vec{F_{L}}$ in Scheme 1 is opposite to the motion of the rotor, since for maintaining the global charge neutrality, the relative displacement is the most important, that means either the rotor is static and the liquid moves in the direction of $\vec{F_{L}}$ or the rotor moves with respect to the liquid in the opposite direction. Under these conditions, a maximum angular displacement of ≈50° with an angular speed of ≈12.5°s^−1^ was recorded. At this angle, motion completely stops, due to a decrease of the polarization potential difference along the BPE, which translates into a decrease of the proton flux and the concomitant Lorentz force. According to the principles of BE, the highest *ΔV* is generated when the BPE is oriented parallel to the applied electric field. Consequently, since in this position the highest ionic flux is produced, the magnitude of the Lorentz force related to the redox reactions is higher than the macro‐MHD convection of the feeder electrodes, causing the rotational displacement of the polarized object. However, when the BPE rotates, *ΔV* decreases as the angle (*α*) between the electric field lines and the object's main axis increases, according to

(1) ΔV=εlcosα

Such a lower *ΔV* value leads to a proportional decrease of the proton flux and its associated $\vec{F_{L}}$, until the induced propulsion force is comparable in magnitude with the macro‐MHD convection, thus canceling both opposing forces. To provide a more quantitative understanding of this phenomenon, plots of the angular acceleration and the theoretical *ΔV* as a function of the rotation angle were analyzed (Figure S2, Supporting Information). The angular acceleration was used as a direct indication of the magnitude of the magnetoelectrochemically induced Lorentz force and was estimated by evaluating the change of angular speed as a function of time. As expected, the maximum value of the angular acceleration (≈10°s^−2^) is found when α = 0° (i.e., the axis of the BPE is aligned with the electric field), when the highest theoretical *ΔV* is induced along the BPE (Figure S2, Supporting Information). Then the acceleration decreases, reaching a minimum value when α ≥ 20°, which correlates with the angle where a steeper decrease of the theoretical *ΔV* is produced (Figure S2, Supporting Information, yellow line). However, the BPE rotor still exhibits a continuous displacement with a relatively constant angular velocity, reaching a maximum of α= 50°, where $\vec{F_{L}}$ is no longer sufficient to overcome the macro‐MHD convection. This set of control experiments provides qualitative and quantitative evidence of the magnetoelectrochemical induction of rotation. In addition, according to the principles of BE, for a given electric and magnetic field intensity, the magnitude of the rotation is directly linked to the concentration of the electroactive species in the solution. Thus, it is possible to assume that the angle or speed of rotation, triggered by this unconventional rotation mechanism, might be used as an optical readout of chemical information in solution.

### 2.3 Influence of the BPE Length and the Electric Field on the Dynamic Behavior

**Figure 1:**
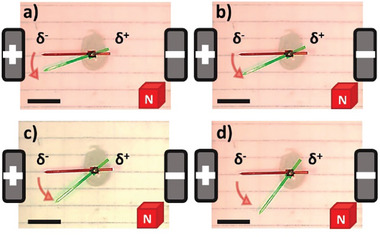
Optical pictures of the dynamic response of a 2 cm long carbon foil rotor for different electric fields a) 0.8 V cm^−1^, b) 1.7 V cm^−1^, c) 3.4 V cm^−1^ and d) 5 V cm^−1^, in the presence of a magnetic field (north pole up). The initial and final positions of the BPE during the wireless rotation are indicated in red and green, respectively. Scale bar 1 cm; readout time for all the experiments is 30 s.

**Figure 2:**
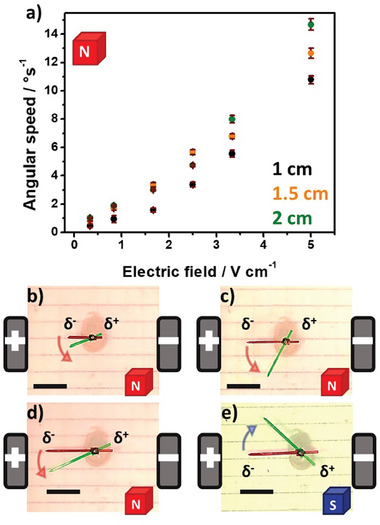
a) Angular speed as a function of the applied electric field obtained in the presence of a magnetic field (north pole up) for BPE having different lengths (indicated in the figure). Optical pictures of the dynamic response of carbon foil rotors with different lengths at a constant electric field (3.4 V cm^−1^): b) *l* = 1 cm, c) *l* = 1.5 cm, and d) *l* = 2 cm in the presence of a magnetic field with the north pole up and e) *l* = 2 cm with the south pole up. The initial and final positions of the BPE during the wireless rotation are indicated in red and green, respectively. Scale bar 1 cm. The readout time for all the experiments is 30 s. The error bars represent the average of three measurements.

After this set of experiments, evaluating globally the wireless magnetoelectrochemical induction of motion, we studied the influence of two fundamental parameters of BE on the dynamic behavior of the rotors: the length of the BPE and the applied electric field. Since the magnitude of $\vec{F_{L}}$, at a constant magnetic field, is directly related to the induced *ΔV* along the BPE, it is reasonable to assume that according to Equation (1), the length of the BPE (*l*) and the applied *ε* have a direct impact on the rotational displacement of the object. First, the dynamic behavior of a 2 cm BPE was evaluated by applying different external *ε* values in the range between 0.3 and 5 V cm^−1^ (Video S2, Supporting Information) Under these conditions, in theory, it is possible to induce a sufficient polarization potential difference (between 0.6 and 10 V) triggering the corresponding redox reactions (Figure S2, Supporting Information). As expected the devices exhibit the characteristic anticlockwise rotation for all *ε* values (**Figure** **1**; Video S2, Supporting Information), originating from the magneto electrochemical induction of $\vec{F_{L}}$. In addition, an increase in the angular speed as a function of the electric field was obtained, corroborating the correlation between the magnitude of *ΔV* and the induced $\vec{F_{L}}$, at a constant $\vec{B}$ value (**Figure** **2**a). A similar tendency was obtained for the BPE with 1 and 1.5 cm length (Figure 2a). However, smaller rotation angles and angular speed values were obtained for the 1 cm length BPE in comparison with the 1.5 and 2 cm devices (Figure 2b−d; Video S3, Supporting Information). This is in good agreement with the principles of BE, where *ΔV* decreases as a function of the size of the BPE. Furthermore, the possible trajectory control was evaluated by changing the orientation of the magnetic field (Figure 2d; Video S3, Supporting Information). By placing the south face of the magnet upward, a clockwise motion was produced, which is predicted by the right‐hand rule. As expected, by fine‐tuning the fundamental experimental parameters of BE (*ε* and *l*) it is possible to modulate the angular speed of the rotor. This is of importance for the possible miniaturization of the device, since according to Equation (1), in order to trigger the rotation, the required electric field increases as the length of the BPE decreases.

### 2.4 Double Electric Field Configuration

**Figure 3:**
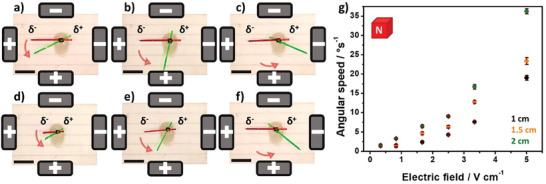
Optical pictures of the dynamic response of a 2 cm long carbon foil rotor for different applied electric fields: a) 0.8 V cm^−1^, b) 1.7 V cm^−1^ and c) 3.4 V cm^−1^, in the presence of a magnetic field (north pole up). Optical pictures of the dynamic response of carbon foil rotors with different lengths at a constant electric field (2.5 V cm^−1^): d) *l* = 1 cm, e) *l* = 1.5 cm and f) *l* = 2 cm in the presence of a magnetic field with the north pole up. The initial and final positions of the BPE during the wireless rotation are indicated in red and green, respectively. Scale bar 1 cm. The readout time for all the experiments is 30 s. g) Angular speed as a function of the applied electric field obtained in the presence of a magnetic field (north pole up) for BPE with different lengths (indicated in the figure). The error bars represent the average of three measurements.

After demonstrating the possible magnetoelectrochemical induction of motion, the next step was to increase the rotation amplitude of the device by using electric fields having two different directions. For this, the feeder electrodes were placed in such a configuration that the two applied electric fields (*ε_1_* ⊥ *ε_2_*) are orthogonal to each other (Scheme S2, Supporting Information) and orientated in the plane of the air/water interface. Theoretically, under these conditions, while *ΔV* induced by *ε_1_* decays during rotation of the BPE, according to *ΔV = ε_1_ l cos α*, the polarization of the BPE increases at the same time due to *ε_2_*, in agreement with *ΔV = ε_2_ l sin α* (Figure S4, Supporting Information). To validate this, we evaluate the dynamic behavior of a 2 cm long BPE at different applied electric fields. It is important to highlight that for each measurement the same electric field value was applied on each pair of feeder electrodes. Once again, an anticlockwise rotation was produced for all the imposed *ε* values (**Figure** **3**a−c; Video S4, Supporting Information). As expected, the simultaneous presence of *ε_2_* produced an additional driving force which leads to a more pronounced rotation. This is demonstrated by a two‐fold increase in the angular speed when using the double feeder electrode configuration. Although the presence of *ε_2_* enhances the motion of the bipolar rotors, it is important to examine whether this effect is due to the spatial distribution of the feeder electrodes or simply originates from the higher global electric field related to the additive effect of *ε_1_* and *ε_2_* (Scheme S2, Supporting Information). In order to provide a suitable explanation, a control experiment with a 2 cm BPE, initially positioned in such a way that an angle of 45° is formed between the object and the electric field lines produced by a single electric field with a twice higher amplitude, was carried out in the presence of a magnetic field (north pole up) (Figure S5, Supporting Information). The experiment was performed in this way to “simulate” the conditions that the rotor experiences when it is exposed to the combination of the two orthogonal electric fields. In this case, the individual electric field vectors should combine and lead to a global electric field with a higher strength. Consequently, this experiment has been carried out with an electric field of 5 V cm^−1^ instead of the 2.5 V cm^−1^, used in the previous experiments, and a starting position of 45° to imitate the position of a rotor aligned with the individual field vector of *ε_1_*. Under these conditions, a maximum angular displacement of ≈100° with an angular speed of ≈25°s^−1^ was obtained. These values are lower in comparison with the ones obtained using the double electric field configurations (160° and 36° s^−1^). This indicates that the improvement in the dynamic behavior is associated with the spatial distribution of the feeder electrodes, as this arrangement allows a smooth and continuous transition of the rotor from a region where *ε_1_* is dominating to an area where *ε_2_* takes over as a driving force.

Furthermore, by comparing the dynamic behavior of three independent BPEs with different lengths at a constant electric field (2.5 V cm^−1^), an increase in the rotation angle as a function of the BPE length was observed (Figure 3e−g; Video S5, Supporting Information). However, the impact of *ε_2_* on the overall trajectory is only significant at electric field values above 2.5 and 1.6 V cm^−1^ for the 1 and 1.5 cm BPEs, respectively. Above these threshold values, a two‐fold enhancement of the maximum angular displacement was produced for all the rotors. From the plot of the angular speed as a function of the applied *ε*, a change in the slope is systematically observed above 2.5 V cm^−1^ for all the BPEs (Figure 3g). Since at this electric field value, the theoretical *ΔV* for all the BPE lengths is above 2 V (Figure S3, Supporting Information), it is reasonable to assume that such a polarization potential triggers additional redox reactions, i.e., the oxidation and reduction of water. This leads to a more pronounced electron and proton flux, from the anode to the cathode of the BPE, which translates into a proportional increase in the intensity of the induced $\vec{F_{L}}$.

**Scheme 2:**
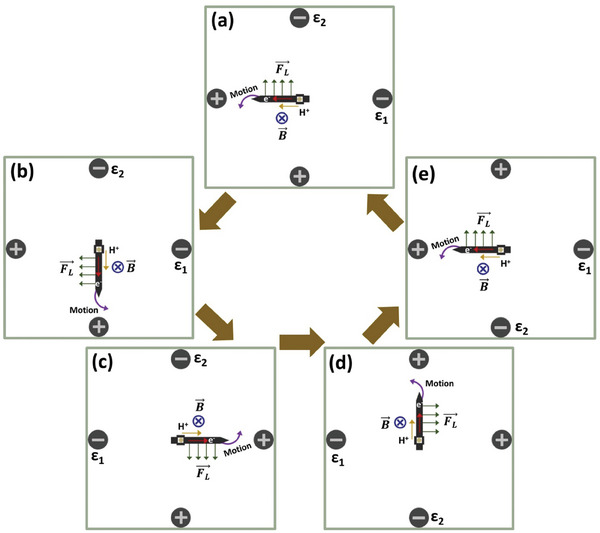
Schematic illustration of the wireless induction of rotation of a carbon strip acting as a BPE in a double electric field configuration with alternating orientation, with a representation of the direction of the electric and charge‐compensating ionic currents, the magnetic field ($\vec{B}$) and the resulting horizontal Lorentz force ($\vec{F_{L}}$).

Although the presence of the additional electric field leads to an enhancement of the global rotational displacement, it is nevertheless limited, despite the BPE being continuously polarized by both electric fields. A possible explanation is that the motion is restricted due to two main phenomena; 1) the competition with the macro‐MHD vortex and 2) the position of the mechanical anchor along the main axis of the BPE. In particular, the latter is of utmost importance since as the BPE rotates, the location of the anchor axis along the rotor changes from being anodic to cathodic and vice versa. As it can be seen in **Scheme** **2** the induced Lorentz force generates a homogeneous lateral flux of fluid along the device, thus the change of relative position of the anchor axis will impact the trajectory of the device. This is a very important aspect, since such a lateral flux is not only the main driving force of the propulsion but constitutes the additional break of symmetry that allows controlling the trajectory of the rotor. Thus, if the anchor is placed on the anodic extremity an anticlockwise motion is predicted, whereas when it is positioned on the cathodic side a clockwise rotation is expected.

### 2.5 Magnetoelectrochemical Full Rotation

Finally, we were interested in testing whether it is possible to produce a full 360° rotation by coupling the double electric field system with an alternating current scheme, like in a classic electric motor. For this, the direction of each electric field was inverted individually, following a defined sequence (Scheme 2). The initial direction of *ε_1_* and *ε_2_*, $\vec{B}$ and the resulting $\vec{F_{L}}$ are depicted in Scheme 2a. Under these conditions, the BPE rotates with an anticlockwise trajectory according to the magnetoelectrochemical induction. Once a 90° angle is reached between the BPE and *ε_1_*, the direction of the latter is inverted (Scheme 2b,c), allowing a half‐turn rotation. Subsequently, once this 180° angle is reached, the direction of *ε_2_* is inverted (Scheme 2c,d). Finally, the complete 360° rotation can be achieved by restoring the initial polarization of *ε_1_* (Scheme 2d,e). In this alternating current scheme, the anchor of the rotor always constitutes the anodic extremity of the BPE, facilitating the continuous motion, due to a globally rotating electric field.

**Figure 4:**
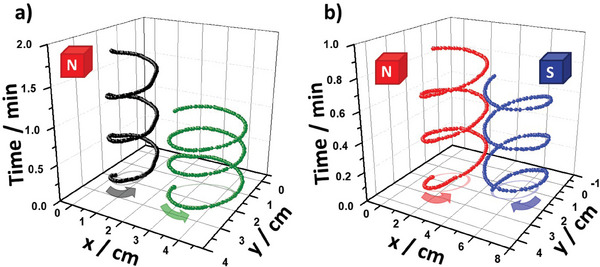
Time‐transient trajectory plot of the carbon foil rotors with different lengths for an electric field of 5 V cm^−1^; a) *l* = 1 cm (black dots) and *l* = 1.5 cm (green dots) in the presence of a magnetic field with the north pole up and b) *l* = 2 cm as a function of the magnetic field orientation, north (red dots) and south (blue dots) pole facing upward.

In this context, we studied the dynamic behavior of three individual BPEs with different lengths, by applying an electric field of 5 V cm^−1^ in the presence of a magnetic field with the north pole facing upward. The direction of each individual electric field was inverted following the above‐mentioned sequence (Scheme 2). Under these conditions, a pseudo‐continuous rotation was generated for all the BPEs (Video S6, Supporting Information). As expected, a linear increase of the angular speed as a function of the length of the BPE was obtained (Figure S6, Supporting Information), which is in agreement with the principles of BE. Plotting the trajectory as a function of time leads to an anticlockwise corkscrew‐type feature for all the BPEs (**Figure** **4**a,b). In addition, a specular dynamic behavior is obtained by changing the orientation of the magnetic field (south pole facing upward), producing a predictable clockwise corkscrew‐type time‐space pattern (Figure 4b blue dots; Video S6, Supporting Information). Furthermore, no significant changes in the angular speed as a function of the orientation of the magnetic field were obtained (Figure S6, Supporting Information, blue and red dots), which indicates the absence of any spin polarization effect.^[^ ^37^, ^38^, ^39^, ^40^ ^]^ The characteristic motion of the BPEs resembles the conventional rotation based on the self‐alignment of magnetic fields commonly used in electric motors. However, in such devices, a direct electric connection is required in order to power a group of spatially distributed electromagnets. In strong contrast, the mechanism proposed here enables the easy and straightforward interconversion of electric energy into kinetic energy via a wireless electrochemical route. Thus, the magnetoelectrochemical rotor can be considered, in a first‐order approximation, as the wet version of a classic electric motor. Furthermore, the rotation of the BPE allows the efficient mixing of solutions, which impacts the mass transport, either from the bulk to the rotor or vice versa. Thus, this concept can be used to design alternative externally driven mixing systems for a variety of applications where mass transport is one of the main concerns, e.g., electrocatalysis or environmental remediation.

## 3 Conclusion

We have demonstrated the possible generation of rotational motion by combining the concepts of bipolar electrochemistry and the magnetically induced Lorentz force. The latter impacts the trajectory of the charge‐compensating ion flux, triggered by the coupled redox reactions, constituting the overall driving force that propels the BPE. Rotational motion is generated by the introduction of an additional break of symmetry via the positioning of the rotor anchor at one of its extremities. In this way, clockwise or anticlockwise motion is triggered as a function of the orientation of the magnetic field. The angle and speed of rotation can be modulated by controlling the length of the BPE, the applied electric field, and, in theory, the strength of the magnetic field. This straightforward mechanism of rotation opens up the possibility to use the amplitude of rotation or the angular speed, e.g., as an analytical tool for the qualitative and quantitative evaluation of redox information. Furthermore, the induced full rotation can be also useful in this context to ensure a sufficient and higher flux of analyte to the electrode/electrolyte interface.

An amplification effect of the rotation was observed when using a double electric field set‐up. Full rotation was achieved by taking advantage of the double electric field system combined with an alternating current scheme. The synergy between the orientation of the magnetic field and the direction of the global electric field allows controlling the overall trajectory of the rotor, producing a predictable clockwise or anticlockwise corkscrew‐type time transient. This set of proof‐of‐concept experiments illustrates the first example of an electrochemical version of an electric motor, where the conventional rotation mechanism based on the self‐alignment of magnetic fields is replaced by wireless magnetoelectrochemical induction. Such an easy and straightforward mechanism for generating rotation might allow the design of macro‐ and micro‐machines where electric energy, combined with an external magnetic field, is directly transformed into kinetic energy in an aqueous environment. In fact, the possible fine‐tuning of the coupled redox reactions at the extremities of the rotors constitutes an additional degree of versatility of the proposed mechanism in comparison with alternative existing concepts, involving gradients of surface tension,^[^ ^4^ ^]^ or external stimuli such as light,^[^ ^8^ ^]^ and sound,^[^ ^10^ ^]^ since it is not limited to a single electroactive system. Finally, the continuous motion of the device allows the efficient mixing of solutions, improving mass transport from the bulk to an electrode or vice versa. This feature opens up new opportunities for applications where mass transport is one of the main constraints, e.g., in electroorganic synthesis or in environmental remediation. The synergy between the wireless magneto electrochemical induction of rotation based on alternative redox systems for applications in the fields of electroanalysis and asymmetric heterogeneous synthesis is currently under study.

## 4 Experimental Section

The rotors were designed as follows: a piece of Kapton−carbon foil was cut in a rectangular shape with approximate final dimensions of 0.1 cm width and 1, 1.5, and 2 cm lengths. Afterward one face of the objects was modified by sputtering a thin gold layer to improve the conductivity (12 min, 35 mA). All the bipolar electrochemistry measurements were performed by placing the rotors at the center of a conventional bipolar cell, with the gold‐modified face in contact with an aqueous solution containing LiClO_4_ (5 mm, Sigma‐Aldrich), sodium dodecyl benzenesulfonate (DBS, 1 µm, Sigma‐Aldrich), p‐benzoquinone (5 mm, Sigma‐Aldrich, ≥98%) and hydroquinone (5 mm, Sigma‐Aldrich, ≥99.5%). All aqueous solutions were prepared with deionized water (MilliQ Direct‐Q). The bipolar cell was composed of a reaction chamber, in which the BPE rotor and the reagents are located, separated from the feeder electrodes by two filter paper membranes. Such a separation was used to avoid the interference of undesired products, formed by the feeder electrodes, with the BPE extremities. Pt‐covered glass plates were positioned as feeder electrodes at the extremities of the cell at a distance of 7 cm and a rectangular FeNdB magnet (*B* = 200 mT, area = 98 cm^2^) was placed below the bipolar cell. All experiments were carried out in three replicates and the error bars represent the standard deviation. Motion was monitored by using a CCD camera (CANON EOS 70D, Objective Canon Macro Lens 100 mm 1:2.8). Images and videos (AVI encoding) were processed with ImageJ software and finally converted to MP4 format. Each video can be considered as an accumulation of frames resulting in a stack projection. The tracking of the rotor motion was performed with the plugin Manual tracking of the ImageJ software.

## Conflict of Interest

The authors declare no conflict of interest.

## Supporting information

Supporting Information

Supplemental Information

Supplemental Information

Supplemental Information

Supplemental Information

Supplemental Information

Supplemental Informarion

## Data Availability

The data that support the findings of this study are available from the corresponding author upon reasonable request.
