# Supplementary material for: Wireless Magnetoelectrochemical Induction of Rotational Motion
Source: Adv Sci (Weinh). 2023 Dec 21;11(9):2306635. doi: 10.1002/advs.202306635 (PMC10916613; doi:10.1002/advs.202306635)
Supplement: Supplementary file 1 — Supporting Information [file ADVS-11-2306635-s002.pdf]

ADVANCED SCIENCE
Open Access

Supporting Information

for *Adv. Sci.*, DOI 10.1002/advs.202306635

Wireless Magnetoelectrochemical Induction of Rotational Motion

Kostiantyn Tieriekhov, Neso Sojic, Laurent Bouffier, Gerardo Salinas and Alexander Kuhn**

Supporting Information

Wireless magnetoelectrochemical induction of rotational motion

Kostiantyn Tieriekhov, Neso Sojic, Laurent Bouffier, Gerardo Salinas and Alexander Kuhn**

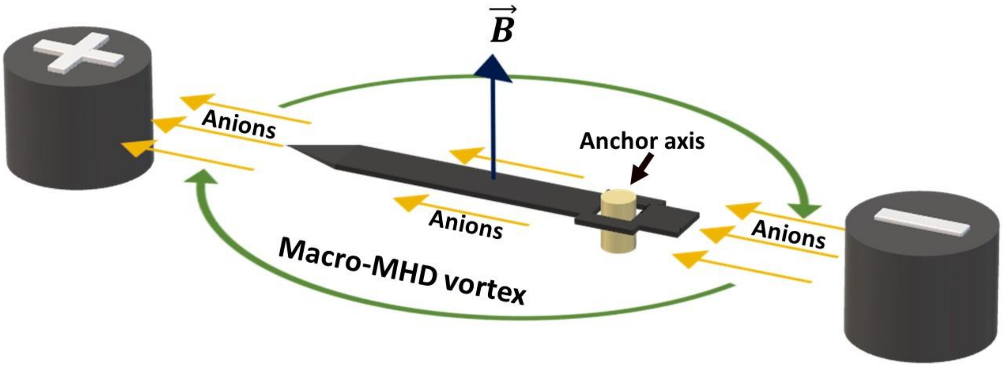

Scheme S1. Illustration of the induction of the macro-MHD vortex (green arrows) associated with the Lorentz force experienced by the electrolyte ions moving between the feeder electrodes.

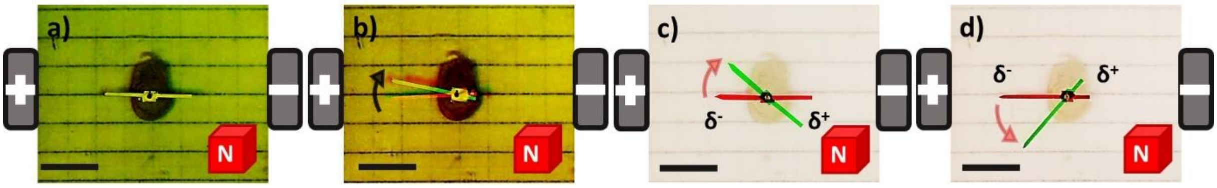

Figure S1. Optical pictures of the dynamic response of four independent rotors with a length of 1.5 cm; two plastic devices with the anchor positioned either (a) at the center or (b) at one extremity, and two carbon foil BPEs with the anchor positioned either at (c) the center or (d) the anodic side at constant applied electric field (5 V cm^{-1}), in the presence of a magnetic field (north pole up). Initial and final positions of the objects during the wireless rotation are indicated in red and green, respectively. Scale bar 1 cm. The readout time for all the experiments is 30 seconds.

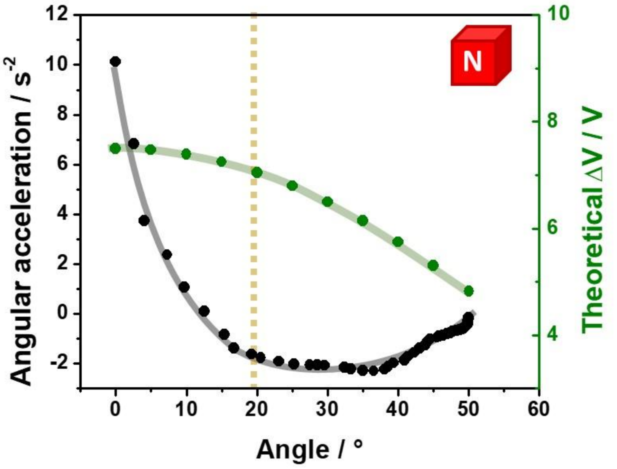

Figure S2. Angular acceleration (black curve) and theoretical polarization potential (green curve) as a function of the angle of rotation, for a 1.5 cm long carbon strip at a constant applied electric field (5 V cm^{-1}), in the presence of a magnetic field (north pole up).

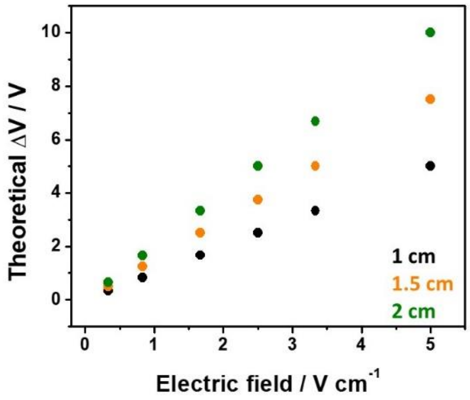

Figure S3. Theoretical polarization potential as a function of the applied electric field for carbon strip rotors with different lengths (indicated in the figure).

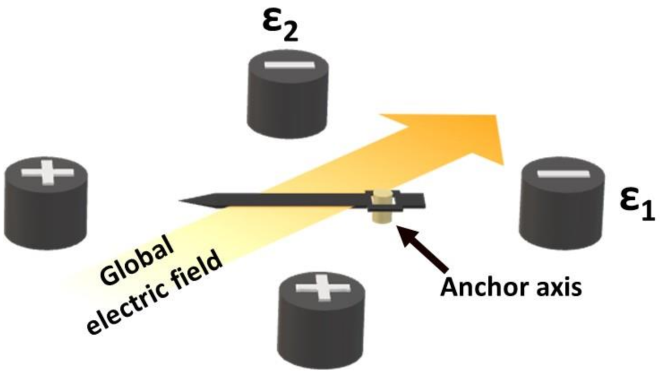

Scheme S2. Schematic illustration of the double electric field set-up used for the magnetoelectrochemical induction of rotation with a representation of the direction of ε_1, ε_2 and the resulting global electric field.

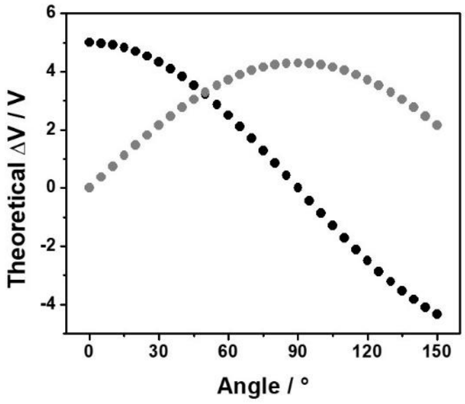

Figure S4. Theoretical polarization potential as a function of the angle of rotation, for a 2 cm long BPE at a constant applied electric field (2.5 V cm^{-1}), calculated with the cosine (black dots) or the sine (gray dots) of the angle.

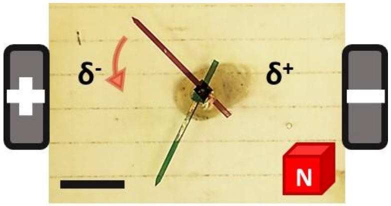

Figure S5. Optical picture of the dynamic response of a 2 cm long BPE, initially positioned in such a way that a 45° angle is formed between the object and the applied electric field (5 V cm^{-1}), in the presence of a magnetic field (north pole up). Initial and final positions of the rotor are indicated in red and green, respectively. Scale bar 1 cm. The readout time for all experiments is 30 seconds.

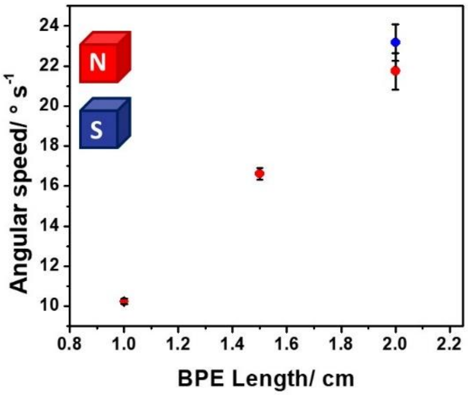

Figure S6. Angular speed as a function of the BPE length obtained during the full magnetoelectrochemical rotation, at an applied electric field of 5 V cm^{-1}. The red and blue dots indicate the north or south pole of the magnetic field, respectively. The error bars represent the average of three measurements.

Video S1. Dynamic behavior of four independent BPEs with different composition and anchor position in the presence of a magnetic field (North pole up, 5x accelerated).

Video S2. Dynamic behavior of a 2 cm BPE as a function of the applied electric field in the presence of a magnetic field (North pole up, 5x accelerated).

Video S3. Rotation as a function of the BPE length in the presence of a magnetic field (5x accelerated).

Video S4. Dynamic behavior of a 2 cm BPE as a function of the applied ε in a double electric field set-up in the presence of a magnetic field (North pole up, 5x accelerated).

Video S5. Rotation as a function of the rotor length in a double electric field system in the presence of a magnetic field (North pole up, 2.5x accelerated).

Video S6. Rotation as a function of the BPE length in a double electric field system with an alternating current scheme in the presence of a magnetic field (7x accelerated).
